# The Connexion of Natural and Divine Truth; or, the Study of the Inductive Philosophy Considered as Subservient to Theology

**Published:** 1838-04

**Authors:** 


Art. VI.—*The Connexion of Natural and Divine Truth; or, the Study of the Inductive Philosophy considered as subservient to Theology.* By the Rev. Baden Powell, m.a. f.r.s. f.g.s., Savilian Professor of Geometry in the University of Oxford.—*London,* 1838. 8vo. pp. 313.

We notice this very interesting and excellent work in connexion with some of the topics discussed in our first article, which was in print before the publication of this volume. The views entertained by the author respecting the study of the value of *final causes* in physiology correspond precisely with those which we have advocated, (see p. 338,) although expressed in different language; our aim having been to demonstrate the injurious tendency of relying upon them in the prosecution of physiology as a science; whilst Professor Powell's object is to show their invalidity as a ground of theological argument, compared with that which is afforded by the study of general laws established independently of them.

"Let us guard," he says, "against the manifest error of taking for granted the thing to be proved. If in physiological, as in any other enquiry, we set out by *assuming* design, intention, or, in a word, a *moral cause,* then it is clear that any *inference* we may wish to draw in support of the truth of natural theology is vitiated and nullified. Considered, therefore, as taken in the correct order of reasoning, the caution expressed by Geoffroy St. Hilaire appears to me strictly just, and the censure which has been cast upon it undeserved. If he did *set out* by 'ascribing intention to the Deity,' he *could not* arrive at any *proof* of such intention. . . . To assign to animals 'a part to play,' before we have traced their analogies, is surely premature in the order of just reasoning, if by that expression anything more be meant than the mere guiding conjecture which the structure of their organs may suggest." (P. 131.)

After adverting to the inferences which may be deduced from the study of individual instances of design on the one hand, and from general laws on the other, Professor Powell continues:

"Either way, then, in the study of nature, there is an equally clear manifestation of that infinite intelligence, which, after the inductive examination of the laws or adjustments in either case, we are directly led to acknowledge as the irresistible conclusion. And, as this is the case with either species of investigation singly, so will it be more preeminently true when both are pursued jointly, as they assuredly may be, without the smallest detriment to each other or confusion of first principles, provided only we keep strictly to the simplest rule of all just reasoning, and do not confound our final conclusion with our first assumption. The highest philosophy is most disposed to cherish a readiness to perceive and admit the fair indications of design and intelligence, in whatever form they may present themselves; and there is a wider expansion given to our views when we thus include the contemplation of order and symmetry (even though we perceive not their end or object) in our notion of design. In such considerations we may find the loftiest exercise of truly philosophical reflections; we shall realize the highest aim of scientific speculation." (P. 136.)

In illustration of these views, Professor Powell adverts to the doctrines of Vegetable Morphology, to which we adverted for the same purpose; and thus closes his argument:

"The conclusion appears to me irresistible, that symmetry of arrangement is as decided a proof of design as adjustment of mechanism; beauty and harmony as clear indications of mind as combination of mechanical action." (P. 140.)

We cannot but rejoice that this subject has been placed in so correct a position by one whose profound acquaintance with the physical sciences, logical correctness of argument, and fearless advocacy of what he deems the truth, entitle his opinions to the calm and impartial consideration of those whose antiquated prejudices they oppose.

